# Ablation of both Cx40 and Panx1 results in similar cardiovascular phenotypes exhibited in Cx40 knockout mice

**DOI:** 10.1042/BSR20182350

**Published:** 2019-02-27

**Authors:** Nicole M. Novielli-Kuntz, Meghan Jelen, Kevin Barr, Leon J. DeLalio, Qingping Feng, Brant E. Isakson, Robert Gros, Dale W. Laird

**Affiliations:** 1 Department of Anatomy and Cell Biology, University of Western Ontario, London, Canada; 2 Robert M. Berne Cardiovascular Research Center, University of Virginia School of Medicine, Charlottesville, VA, U.S.A.; 3 Department of Physiology and Pharmacology London, ON, Canada; 4 Robarts Research Institute, Schulich School of Medicine & Dentistry, University of Western Ontario, London, ON, Canada

**Keywords:** connexin, gap junctions, Pannexin 1

## Abstract

Connexins (Cxs) and pannexins (Panxs) are highly regulated large-pore channel-forming proteins that participate in cellular communication via small molecular exchange with the extracellular microenvironment, or in the case of connexins, directly between cells. Given the putative functional overlap between single membrane-spanning connexin hemichannels and Panx channels, and cardiovascular system prevalence, we generated the first Cx40^−/−^Panx1^−/−^ mouse with the anticipation that this genetic modification would lead to a severe cardiovascular phenotype. Mice null for both Cx40 and Panx1 produced litter sizes and adult growth progression similar to wild-type (WT), Cx40^−/−^ and Panx1^−/−^ mice. Akin to Cx40^−/−^ mice, Cx40^−/−^Panx1^−/−^ mice exhibited cardiac hypertrophy and elevated systolic, diastolic, and mean arterial blood pressure compared with WT and Panx1^−/−^ mice; however assessment of left ventricular ejection fraction and fractional shortening revealed no evidence of cardiac dysfunction between groups. Furthermore, Cx40^−/−^, Panx1^−/−^, and Cx40^−/−^Panx1^−/−^ mice demonstrated impaired endothelial-mediated vasodilation of aortic segments to increasing concentrations of methacholine (MCh) compared with WT, highlighting roles for both Cx40 and Panx1 in vascular endothelial cell (EC) function. Surprisingly, elevated kidney renin mRNA expression, plasma renin activity, and extraglomerular renin-producing cell populations found in Cx40^−/−^ mice was further exaggerated in double knockout mice. Thus, while gestation and gross development were conserved in Cx40^−/−^Panx1^−/−^ mice, they exhibit cardiac hypertrophy, hypertension, and impaired endothelial-mediated vasodilation that phenocopies Cx40^−/−^ mice. Nevertheless, the augmented renin homeostasis observed in the double knockout mice suggests that both Cx40 and Panx1 may play an integrative role.

## Introduction

The connexin (Cx) and pannexin (Panx) families of large-pore forming channel proteins facilitate the passage of various ions, metabolites, and signaling molecules between cellular cytoplasms, either through extrusion into the extracellular milieu, or in the case of connexins, directly between cells [1,2]. Connexins may functionally overlap with the activities of pannexin channels and participate in intercellular signaling by generating functional hemichannels at the cell surface, although this is not well documented *in vivo* [3–5]. Conversely, the most well-understood pannexin, pannexin1 (Panx1), has been demonstrated to form large-pore membrane channels, which facilitate autocrine/paracrine-mediated signaling via the release of purine nucleotides, most notably ATP [6]. Within the mammalian cardiovascular system (cardiac tissue and peripheral vasculature) connexins and Panx1 participate in both protein-specific and homologous protein functions that coordinate cellular responses requisite for vascular homeostasis. The enrichment of both proteins within the same cardiovascular tissues suggests a functional co-operation between connexins and Panx1; however, it is not clear whether Panx1 plays any additive or synergic role [7–9].

In the mammalian heart, connexins are obligatory for normal myocardial and vascular development and function [10]. The synchronized contraction of myocardial tissue, as well as the conduction of electrical impulses generated by the sinoatrial (SA) node relies on gap junctional intercellular communication—primarily via Cx43, Cx40, and Cx45 isoforms [11]. Generally, Cx45 expression remains confined to the SA node and atrioventricular node; however, the Bundle of His and Purkinje fibers express Cx45, Cx40, and Cx43 [12]. Interestingly, the Cx40 isoform, which has a well-established role in regulating blood pressure and renal-renin secretion [13], is developmentally regulated in the murine heart. Peak expression levels are observed ubiquitously throughout fetal cardiac tissue at E14, only later to be confined in the atria tissue and the conduction system of the adult heart, while Cx43 remains highly expressed throughout the heart [14]. Human mutations in the gene encoding Cx40, *GJA5*, have been reported to cause atrial fibrillation [15], and the deletion of Cx40 in murine models results in myocardial hypertrophy, fibrosis, arrhythmia, and conduction disturbances (i.e. increased P-wave and QRS duration) [16]. In the vasculature, Cx37, Cx40, Cx43, and Cx45 are found in endothelial and smooth muscle cells (ECs and SMCs), but exhibit distinct expression profiles depending on the cell type and position in the vascular tree (i.e. artery to arteriole) [17]. In particular, Cx40 expression in the endothelium is critically important for maintaining vascular homeostasis through EC–EC gap junctions and EC–SMC myoendothelial gap junctions [18]. Cx40 null mice exhibit deficits in EC-mediated vasodilation and conducted vasodilatory responses along arteriolar segments [9]. Thus, Cx40 appears to play a complex role in the cardiovascular system.

In addition to Cx40, Panx1 has also emerged as a regulator of cardiovascular function through the regulated release of intracellular ATP from the vascular wall [7,19–22]. A predominant role for this large-pore protein channel has been established in the regulation of vascular tone [23–27] and α1-adrenergic receptor-mediated vasoconstriction in mice and humans [7,28,29]. In this way, ATP acts as a signaling molecule that promotes purinergic signaling within cardiovascular tissues [24,30]. Notably, the role of Panx1 channels has not been well characterized in the healthy heart. Early studies using *in vitro* rat cardiomyocyte culture have implicated that Panx1 functions at the cell surface as a calcium-sensitive large conductance cation channel [31], and that Panx1 genetic ablation promotes cardiac electrophysiological abnormalities (prolonged depolarization/repolarization and atrial fibrillation susceptibility) [32]. In cardiac inflammation and ischemia models, Panx1-mediated ATP release plays a pathological role in cardiac fibrosis, but a cardioprotective role against ventricular infarct size in mice [33–36]. While pannexin isoforms 2 and 3 (Panx2 and Panx3) have been identified in a small subset of vascular tissue within the murine arterial network [21], it has been reported that cardiac tissue expresses little Panx2 that is intracellularly localized, and no Panx3 [37,38]. Thus, primarily Panx1 channels participate in a myriad of processes within the vasculature and potentially the heart to support healthy organ function.

Although Cx40 and Panx1 originate from distinct protein families, both appear to play critical roles in the heart and vasculature. It is not known however, whether compensation, redundancy, or unique roles exist for Cx40 and Panx1 in supporting cardiovascular function. To address this question we developed the first mouse line lacking both Cx40 and Panx1 (Cx40^−/−^Panx1^−/−^) and we hypothesized that deletion of Panx1 in Cx40-deficient mice would exacerbate cardiac phenotypes observed in Cx40^−/−^ mice. In the current study, we found that Cx40^−/−^Panx1^−/−^ mice are viable, fertile, and exhibit similar adult morphological development to wild-type (WT) mice. Compared with WT and Panx1^−/−^ mice, Cx40^−/−^Panx1^−/−^ mice exhibit cardiac hypertrophy, and significantly elevated arterial blood pressure that phenocopies Cx40^−/−^ mice. Furthermore, aortic ring myography revealed reduced endothelium-dependent vasodilation in all tested genotypes compared with WT. Interestingly, Cx40^−/−^Panx1^−/−^ mice demonstrated significantly elevated kidney renin mRNA and plasma renin activity, surpassing the elevated levels observed in Cx40^−/−^ mice, and greater ectopic populations of juxtaglomerular renin-producing cells within extraglomerular regions. These results demonstrate that Panx1 ablation does not augment cardiac effects observed in mice lacking Cx40, suggestive of independent functions for these two channel proteins in cardiac development and function. However, in the vasculature and the kidney, our data suggest a potential relationship between Cx40 and Panx1 function within the endothelium, and the phenotypic status of renin-producing cells in juxtaglomerular and extraglomerular regions.

## Experimental procedures

### Engineering and characterization of mice

Cx40^−/−^Panx1^−/−^ mice were bred in-house from Cx40^−/−^ and Panx1^−/−^ single knockout mice that had been previously backcrossed on to a C57BL/6N background for approximately six and four generations, respectively. Due to reported sex-related effects on the cardiovascular system, development of cardiovascular disease, and renin–angiotensin regulatory mechanisms [39–47], we used male mice for the current study. The Cx40 knockout mouse was generated by Simon et al. [40] and was a generous gift from Dr. David Paul via Dr. Donglin Bai (University of Western Ontario, London, ON). Panx1 null mice were kindly provided by Dr. Vishva Dixit at Genentech and have been previously characterized by Qu et al. [41]. Male WT mice of the C57BL/6N strain from Charles River were used as controls. Mice were housed four per cage, received food and water *ad libitum*, and were maintained on a 12-h light/dark cycle at 24°C. PCR genotyping was performed to ensure the ablation of Cx40 and Panx1 as described previously using ear clip DNA as a template [41,42]. For the characterization of male mouse weight/size, knockout and WT mice from three to six litters, with a minimum of ten mice per time point, were weighed periodically for up to a year. Unless otherwise indicated, data were collected from adult mice at 3–5 months of age. For experimental end points (i.e. during tissue collection), all animals were killed via cervical dislocation and organs were fixed in formalin or flash-frozen and stored at −80°C. All studies performed were in accordance with the Animal Care Committee of Western University.

### q-PCR analysis

RNA was extracted using the Qiagen RNeasy kits (Qiagen) from the atria and ventricle of 3-month-old WT and Panx1^−/−^ mice. cDNA was synthesized using the first-strand cDNA synthesis kit (SuperScript VILO). Panx1 transcript levels were determined using mouse Panx1-specific primers (5′-ACAGGCTGCCTTTGTGGATTCA-3′; 5′-GGGCAGGTACAGGAGTATG-3′) and the PowerUp SYBR Green Mastermix (Life Technologies) in a Bio-Rad CFX96 real-time system. Results were normalized to 18S rRNA. Brain tissue was used as a positive control, and tissues from Panx1^−/−^ mice were included with WT tissue as a negative control; *n*=3 per group.

### Litter size characterization

Sizes of WT and knockout mice were tracked by setting up multiple breeder cages for each strain and counting the number of offspring per litter up to 3 days post-natal at the time of collecting genotyping samples (number of litters from a total of 10–17 dams encompassing all genotypes), WT, *n*=30; Cx40^−/−^, *n*=29; Panx1^−/−^, *n*=50; Cx40^−/−^Panx1^−/−^, *n*=15).

### Heart and kidney weight characterization

Three weeks and 3-month old mice were killed via cervical dislocation, and their hearts and kidneys excised. Organs were exsanguinated, rinsed in PBS, and dried off excess solution on Kimwipes. Dry heart and kidney weights were then recorded and normalized to the initial mass of the animal to obtain a normalized organ weight (mg/g, *n*=6 hearts per group).

### Western blot analysis

Tissue lysates of knockout and WT hearts were prepared via homogenization of atria or ventricles on ice in lysis buffer (150 mM NaCl, 1 mM EDTA, 1 mM EGTA, 1% Triton X-100, 10 mM Tris/HCl) with protease and phosphatase inhibitors (Roche-Applied Sciences; 100 mM NaF and 100 mM Na_3_VO_4_). Thirty micrograms of protein from lysates were resolved on a 10% SDS/PAGE gel when probing for Cx40 and Cx43 or a 4–20% gradient gel (Bio-Rad, Hercules, CA) when probing for N-cadherin, collagen I, and fibronectin, and then transferred to a nitrocellulose membrane using an iBlot Dry Blotting system (Invitrogen). Membranes were blocked in a 3% BSA/0.05% Tween-20 PBS solution (PBST) for 30 min at room temperature. Membranes were then probed using the following primary antibodies: rabbit anti-Cx43 (1:5000, Sigma, C 6219); goat anti-Cx40 (1:500, Santa Cruz Biotechnology, sc-20466); mouse anti-collagen 1 (1:500, Abcam, ab90395), mouse anti-fibronectin (1:500, Abcam, ab23750), mouse anti-N-cadherin (1:200, BD Signal Transduction, 610920), and mouse anti-GAPDH (Santa Cruz Biotechnology, sc-365062), diluted in blocking solution at 4°C overnight. Membranes were then washed three times for 5-min intervals with PBST and incubated with fluorescently tagged secondary anti-rabbit Alexa Fluor® 680 (1:5000, LI-COR Biosciences, ab175772) or anti-mouse IRdye 800 (1:5000, Rockland Immunochemicals, Inc., 610-132-003). Protein expression and densitometry analysis was determined using Odyssey Infrared Imaging System and software (LI-COR Biosciences). Samples were normalized to GAPDH loading controls; *n*=3 samples per group.

### Immunofluorescence microscopy

Immunofluorescence was performed as previously described [43]. Briefly, 10% formalin-fixed paraffin embedded heart and kidney sections (6 μm) were deparaffinized, incubated in Sodium Citrate Buffer (10 mM, 0.05% Tween-20, pH 6.0) for 30 min at 90–100°C, and washed with PBS. For cryosections (10 μm), hearts were fixed in formalin fixative overnight at 4°C and immersed in 30% sucrose cryoprotection solution prior to sectioning. Sections were blocked in 3% BSA/0.1% Triton X-100 for 1 h at room temperature. Sections were probed overnight at 4°C using the following primary antibodies: rabbit anti-Cx43 (1:500, Sigma, C 6219), goat anti-Cx40 (1:200, Santa Cruz Biotechnology, sc-20466), mouse-anti N-cadherin (1:300, BD Signal Transduction, 610920), or goat-anti mouse renin (1:200, R&D Systems AF4277). Secondary Alexa Fluor® 555–conjugated anti-rabbit, anti-mouse, or anti-goat (1:500, Invitrogen Molecular Probes, A21425, A21429 or A21431) and Alexa Fluor® 488–conjugated anti-rabbit or anti-mouse (1:500, Invitrogen Molecular Probes, A11008 or A11017) antibodies were applied for 1 h at room temperature to detect primary antibody binding. Hoechst counterstain was applied for a 10-min period to label nuclei (1:10000, Invitrogen Molecular Probes, H3570). Coverslips were mounted with Airvol and imaged using a Zeiss LSM 800 confocal microscope and Zen image capture software (63× oil immersion objective or 40× water immersion objective).

### Quantitation of cardiomyocyte area

Formalin-fixed left atria and ventricles of WT, Cx40^−/−^, Panx1^−/−^, and Cx40^−/−^Panx1^−/−^ mice were sectioned (6 µm), respectively, deparaffinized, and stained with Wheat Germ Agglutinin (1:10000 in PBS, Thermo Fisher, W11261), to visualize sarcolemma membranes [44]. Airvol was used to mount coverslips and specimens were imaged using the Zeiss LSM 800 confocal microscope and Zen capture software using a 40× water immersion objective. Images were imported to ImageJ and converted into a binary image so that edges of cardiomyocytes could be identified. Cardiomyocyte area was calculated using the ImageJ area tool, and the area of 15 cardiomyocytes for four regions of interest, for each section over two tissue sections at least 24 µm apart were quantitated and averaged for each animal.

### Cardiac fibrosis assessment

Whole hearts were sectioned longitudinally for all mouse genotypes (7 μm), deparaffinized, and stained with Masson’s Trichrome using standard procedures to analyze the extent of cardiac fibrosis. Bright field microscopy was used to obtain images from the left atrial and left ventricular wall and all images were analyzed for interstitial and perivascular collagen deposition [45].

### Arterial blood pressure recording

Arterial blood pressure was recorded as described previously [46]. Blood pressure was assayed in mice 3–5 months of age at the beginning of the day. Prior to surgical procedures, mice were anesthetized using isofluorane/O_2_, where lack of hind limb reflex to toe-pinch indicated sufficient plane of anesthesia. Mice were placed in supine position and maintained under anesthesia via a nose cone. Body temperature was monitored using a rectal temperature probe and maintained at 37°C using an electric heating pad. Briefly, a small incision was made in the neck and the right carotid artery was isolated and cannulated using a Millar-tip transducer catheter (model SPR-261, 1.4F; Millar Instruments, Inc., Houston, TX). The catheter was advanced toward the right atria and secured in place so that steady arterial blood pressure signal was obtained. A 10–15-min acclimatization period was performed prior to experimental recording. Blood pressure and calculated variables (systolic and diastolic blood pressure, mean arterial pressure, and heart rate) were recorded and analyzed using Lab Chart 7 software (AD Instruments, Colorado Springs, CO, U.S.A.; *n*=4–6 per animal group).

### Mouse echocardiography

Mouse echocardiography was performed as previously described [47]. M-Mode echocardiography was used to assess left ventricle (LV) wall thickness, LV internal dimension, LV ejection fraction, and LV fractional shortening. Using the Vevo 2100 ultrasound imaging system and a 40-MHz probe, 2D images of the LV were captured in the short-axis view. The M-Mode cursor was positioned perpendicular to the anterior and posterior LV walls. Systolic and diastolic LV thickness (anterior and posterior wall), LV internal end-diastolic dimension (LVIDd) and LV internal systolic dimension (LVIDs) were measured from M-mode recordings. All dimension measurements were calculated as an average over three cardiac cycles, per animal. Ejection fraction was calculated using the following equation: EF (%) = [(LVIDd)^3^ − (LVIDs)^3^]/(LVIDd)^3^ * 100. Fractional shortening was calculated as: FS (%) = (LVIDd − LVIDs)/LVIDd * 100.

### Kidney renin mRNA

Kidneys were homogenized in TRIzol reagent (Thermo Fisher Scientific, Waltham, MA, U.S.A.). The aqueous layer formed after chloroform addition was purified for RNA using an RNeasy kit (Qiagen, Germantown, MD, U.S.A.) with DNase treatment. cDNA was synthesized using Superscript VILO (Thermo Fisher Scientific, Waltham, MA, U.S.A.) with identical RNA inputs for all samples. qPCR was performed on cDNA using PowerUp Sybr Green Master Mix (Thermo Fisher Scientific, Waltham, MA, U.S.A.) on a Bio-Rad CFX 96 (Bio-Rad, Hercules, CA, U.S.A.; *n*=7–8 per group). Primers were as described by Wagner et al. (2007) [48] for renin: ATGAAGGGGGTGTCTGTGGGG (upper), ATGCGGGGAGGGTGGGCACCT (lower), and β-Actin as an endogenous control: CGGGATCCCCGCCCTAGGCACCAGGGTG (upper), GGAATTAGGCTGGGGTGTTGAAGGTCTCAAA (lower).

### Vasomotor studies

Vascular reactivity of WT, Cx40^−/−^, Panx1^−/−^, and Cx40^−/−^Panx1^−/−^ mouse aortic rings were assessed by performing isometric tension experiments on a wire myograph as previously described [49,50]. Three to four months old mice were killed via cervical dislocation, and thoracic aortas were excised, cleaned, and cut into 2-mm rings. Preparations were mounted in organ bath chambers containing 5 ml of 37°C oxygenated Krebs physiological salt solution (130 mM NaCl, 14.9 mM NaHCO_3_, 10.0 mM glucose, 4.70 mM KCl, 1.17 mM MgSO_4_, 1.18 mM KH_2_PO_4_, 1.60 mM CaCl_2_, and 0.027 mM EDTA). Contractile responses to phenylephrine (PE) (1 nM to 30 μM) and potassium chloride (KCl) (10–100 mM) were recorded in vessels stretched to a passive resting tension of either 1000 or 1250 mg. These conditions were determined from earlier pilot experiments. Similarly, dilatory responses were assessed with either methacholine (MCh) (0.1 nM to 30 μM) or sodium nitroprusside (SNP) (0.01–30 nM) in PE (10 μM) pre-contracted rings.

### Plasma renin measurement

Blood was collected via cardiac puncture with EDTA and centrifuged to isolate plasma (*n*=6–7 per group). For the renin activity assay, 25 μl of plasma was added to 95 μl of buffer (20 mM Tris/Cl, 30 mM EDTA, 0.2% BSA, pH 7.4 at 37°C). Synthetic renin substrate (angiotensinogen, Sigma–Aldrich, Oakville, ON) was added to a final concentration of 1 μM and PMSF protease inhibitor was added to a final concentration of 1 mM. Tubes were incubated on ice to assess native renin via endogenous angiotensin I levels or incubated for 1 h at 37°C to assess active renin levels. Angiotensin I was measured using ELISA as per manufacturer’s instructions (Enzo Life Sciences, Farmingdale, NY).

### Statistical analysis

Results are provided as means ± S.D. One-way ANOVA with a Tukey’s post-test was used to assess differences between genotype means for litter size, heart and kidney weight, cardiomyocyte area, blood pressure and heart rate variables, diastolic and systolic LV internal diameter and ventricle wall thickness, ejection fraction and fractional shortening, kidney renin mRNA, and plasma renin measurements. Differences between genotype means for aortic ring vasoreactivity to increasing concentrations of PE, KCl, MCh, and SNP were assessed using a two-way ANOVA followed by Tukey’s multiple comparisons test. All statistical analyses were performed using Prism 6 (GraphPad, La Jolla, CA). Means were deemed statistically significant when *P*<0.05.

## Results

### Cardiac Panx1 expression and developmental phenotypes of mice lacking Panx1 and/or Cx40

**Figure 1:**
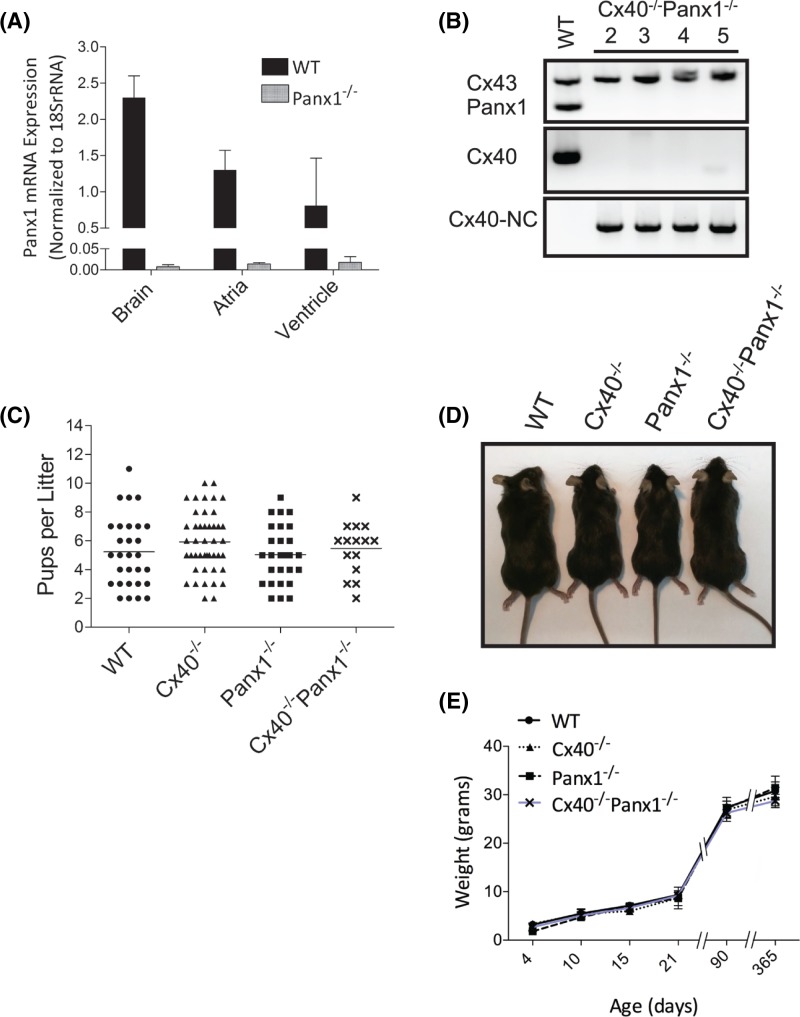
Characterization of Cx40/Panx1 knockout mice (**A**) qPCR revealed that Panx1 was expressed in the heart atria and ventricles, and the brain of WT mice, but absent from Panx1 null mice. (**B**) PCR genotyping confirmed that both Panx1 and Cx40 were ablated in double knockout mice as revealed in lanes 2–5 representing different mice. Cx43 was present in all mice and the insertion of the Cx40 neocassette (Cx40-NC) reaffirmed the ablation of Cx40. (**C**) WT and knockout mouse mean litter size at birth (number of litters from 10–17 dams, WT, *n*=30; Cx40^−/−^, *n*=29; Panx1^−/−^, *n*=50; Cx40^−/−^Panx1^−/−^, *n*=15; Dams). (**D**) Representative photograph revealing that mice of all four genotypes are of similar size. (**E**) Assessment of mouse weight over 1 year revealed that all WT and knockout mice have similar weights and weight gain (*n*=3–6 litters, 10 mice/time point).

To evaluate the levels of Panx1 present in the WT mouse heart, RNA was extracted from atrial and ventricular tissues and subjected to qPCR analysis. Panx1 transcripts were detected in both the atria and the ventricles, but were less abundant than *Panx1* mRNA found in brain tissue. This is consistent with a report demonstrating that Panx1 protein is found in both rodent atrial and ventricular myocytes [31]. No detectable Panx1 transcripts were found in heart and brain tissue taken from Panx1^−/−^ mice (Figure 1A). PCR genotyping revealed that Cx40 and Panx1 were ablated from the Cx40^−/−^Panx1^−/−^ mouse generated from crossing Panx1 null mice with mice lacking Cx40 (Figure 1B). The presence of *Cx43* in control and double knockout mice confirmed the integrity of the samples used in genotyping (Figure 1B). Upon assaying litter sizes, all genotypes demonstrated a similar spread of litter sizes and average number of pups per litter (Figure 1C). To determine whether mice lacking Panx1, Cx40, or both Panx1/Cx40 exhibited any growth changes compared with each other or WT mice, assessment of size and weight over a 1-year period was performed. These measurements revealed no differences amongst the four-mice genotypes (Figure 1D,E).

### Atrial Cx40 distribution and expression is conserved in Panx1^−/−^ mice

**Figure 2:**
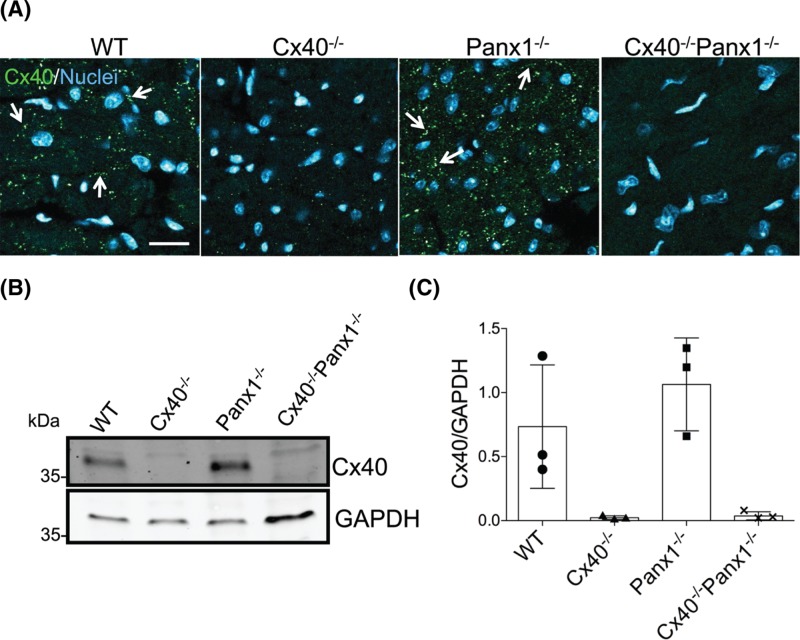
Cx40 localization and expression levels are unchanged in Panx1-ablated mice (**A**) Fluorescent micrographs reveal similar localization of Cx40 (green) in WT and Panx1^−/−^ mouse atria (arrows = Cx40 gap junctions). The absence of Cx40 gap junction plaques in Cx40^−/−^ and Cx40^−/−^ Panx1^−/−^ confirms the ablation of Cx40. (**B**) Western blot and (**C**) quantitation reveal similar levels of Cx40 in WT and Panx1^−/−^ mice, and its ablation in Cx40^−/−^ and Cx40^−/−^Panx1^−/−^ atria (*n*=3). Immunoblotting for GAPDH was used as a loading control. Nuclei in (A) were stained with Hoechst dye. Scale bar = 20 μm.

Cx40 is primarily expressed throughout atrial tissue, thus immunofluorescence was performed in order to determine whether the ablation of Panx1 altered the expression or distribution of Cx40. In the atria of WT and Panx1^−/−^ mice, Cx40 gap junctions revealed a similar localization pattern, while Cx40 was predictably absent from the hearts of Cx40^−/−^ mice (Figure 2A). Western blots further revealed that there was no quantitative change in Cx40 expression levels in Panx1^−/−^ mice (Figure 2B,C) compared with WT. Thus, Cx40 localization and expression is not regulated by the presence or absence of Panx1.

### Cx40^−/−^ and Cx40^−/−^Panx1^−/−^ mouse hearts are hypertrophic

**Figure 3:**
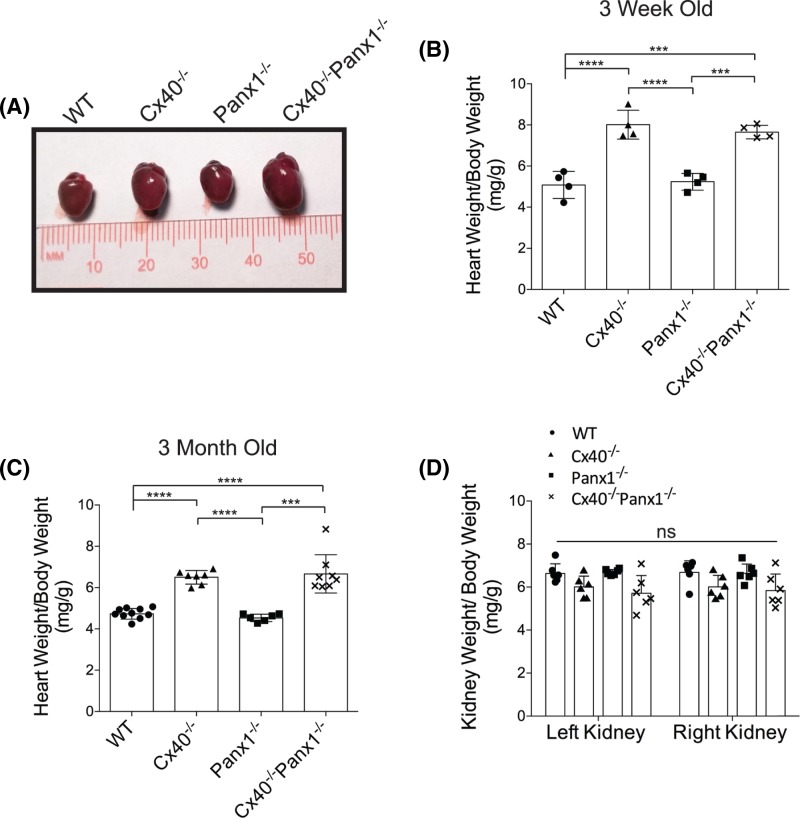
Increased heart mass found in Cx40^−/−^ and Cx40^−/−^Panx1^−/−^ mice (**A**) Photographic examples of representative hearts from 3-month-old mice across all genotypes highlighting the enlarged hearts in Cx40^−/−^ and Cx40^−/−^Panx1^−/−^ mice. When heart weights were examined, it was found that Cx40^−/−^ and Cx40^−/−^Panx1^−/−^ mice had increased heart weight relative to body weight at the ages of (**B**) 3 weeks (*n*=4) and (**C**) 3 months (*n*=6) as compared with WT and Panx1^−/−^ mice. (**D**) Mean kidney weights were compared in 3-month-old mice and found to be similar (*n*=6). ***, *P*<0.001; ****, *P*<0.0001; ns, not significantly different.

**Figure 4:**
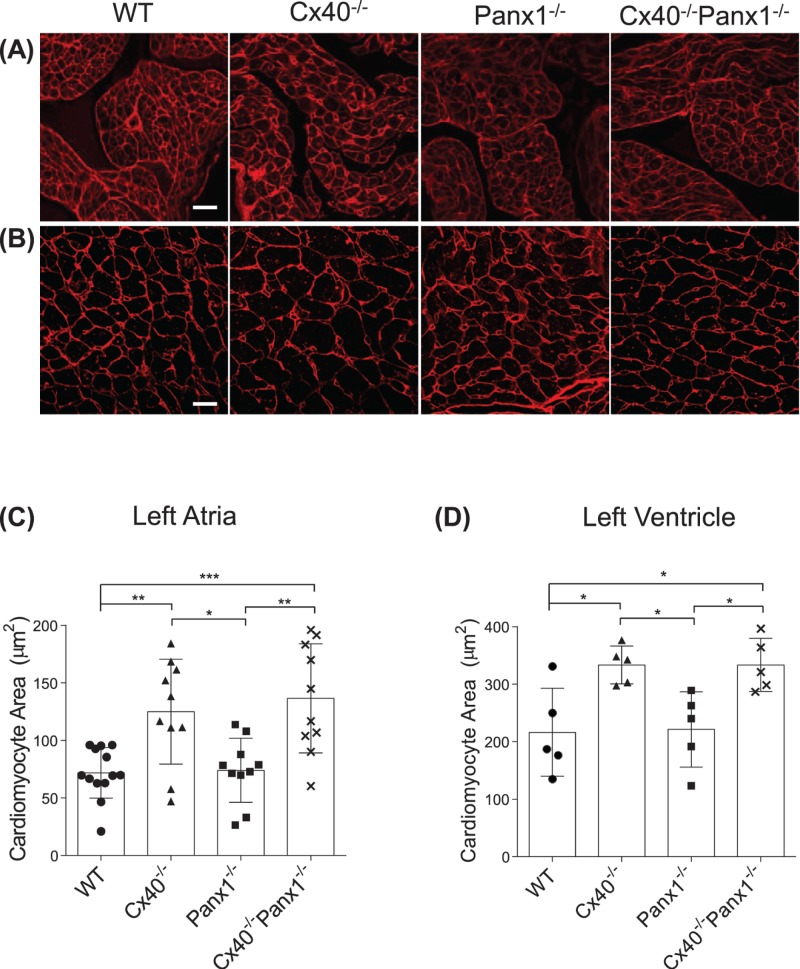
Cx40^−/−^ and Cx40^−/−^Panx1^−/−^ mice have hypertrophic cardiomyocytes Wheat germ agglutinin staining (red) of the cardiomyocyte cell surface in left (**A**) atrial and (**B**) ventricular cross-sections taken from 3-month-old WT and knockout mice. Quantitation of the average cardiomyocyte cell area revealed greater atrial (*n*=10–13; (**C**)) and ventricular (*n*=5; (**D**)) cardiomyocyte size in Cx40^−/−^ and Cx40^−/−^Panx1^−/−^ mouse hearts. *, *P*<0.05; **, *P*<0.01, ***, *P*<0.001. Scale bar = 20 µm.

Given the enlarged appearance of Cx40^−/−^ and Cx40^−/−^Panx1^−/−^ mouse hearts (Figure 3A), it was not unexpected that both genotypes had greater heart mass compared with Panx1^−/−^ and WT mice at 3 weeks (Figure 3B, *P*<0.0001–0.001) and after maturation in 3-month-old adults (Figure 3C, *P*<0.0001–0.001). Left and right kidney weights were also assessed and found to be similar amongst all four genotypes (Figure 3D). To determine if the increased heart mass of Cx40^−/−^ and Cx40^−/−^Panx1^−/−^ mice was due to cardiomyocyte hypertrophy, cardiomyocyte cross-sectional area of left atrial and ventricular tissue was measured (Figure 4A,B). Quantitation revealed that cardiomyocyte area was significantly elevated in the left atria and ventricle of 3-month-old Cx40^−/−^ and Cx40^−/−^/Panx1^−/−^ mice, compared with WT and Panx1^−/−^ (Figure 4C,D, *P*<0.05–0.001). Furthermore, we demonstrated that *in vivo* systolic and diastolic left ventricular thickness was also greater in Cx40^−/−^ and Cx40^−/−^Panx1^−/−^ mice, predominantly the anterior LV wall (see Figure 8B, *P*<0.01–0.05) as measured by M-mode echocardiography.

### Cardiac tissue fibrosis remains minimal in the absence of Cx40 and Panx1

**Figure 5:**
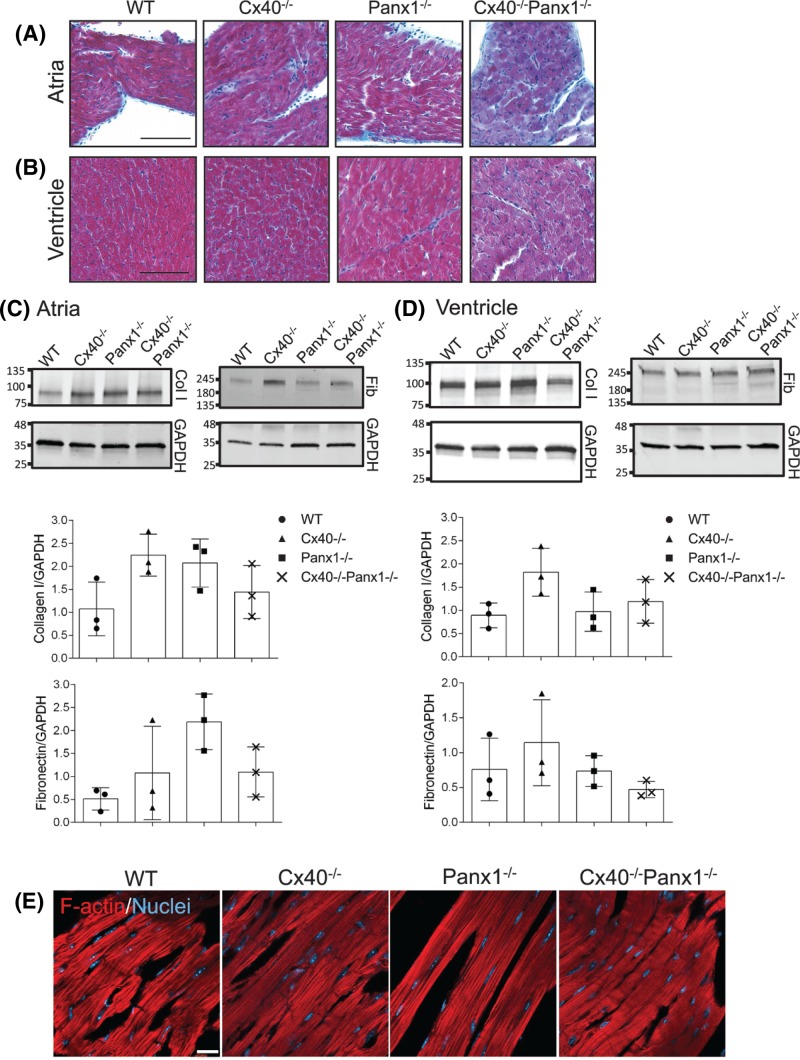
Masson’s Trichrome staining and assessment of extracellular matrix proteins in the hearts of knockout mice Light micrographs of Masson’s Trichrome staining in the left (**A**) atria and (**B**) ventricle of 3-month-old mice revealed minimal fibrosis in all knockout mice compared with WT. Western blotting revealed that the levels of collagen I (col I) and fibronectin (Fib) were similar between mouse genotypes in (**C**) atria and (**D**) ventricle tissue protein lysates (*n*=3). (**E**) Phalloidin (red) was used to visualize F-actin in the ventricle of WT and knockout mice. Immunoblotting for GAPDH was used as a loading control. Trichrome staining images; scale bar = 40 μm; fluorescent phalloidin images; scale bar = 20 μm.

Cardiac fibrosis in 3-month-old mouse hearts was assessed via Masson’s Trichrome staining and Western blot for various extracellular matrix proteins. Masson’s Trichrome staining of the left atria (Figure 5A) and ventricle (Figure 5B) demonstrated minimal interstitial fibrosis in the hearts of Cx40^−/−^, Panx1^−/−^, and Cx40^−/−^Panx1^−/−^ mice compared with WT. Immunoblotting indicated similar levels of collagen 1 and fibronectin in atria and ventricle homogenates amongst all four genotypes (Figure 5C,D). Finally, myofibril arrangement was evaluated by phalloidin staining (Figure 5E) and F-actin localization revealed that the fiber organization was comparable amongst all four genotypes.

**Figure 6:**
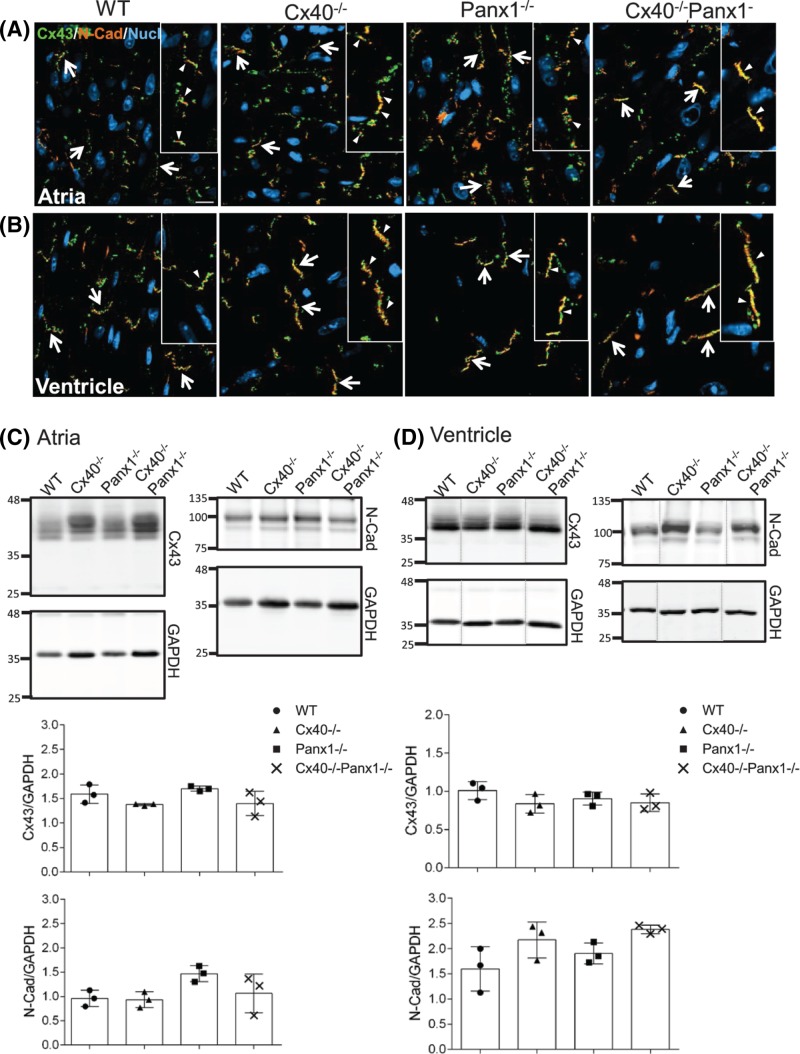
Junctional proteins are unaltered in the hearts of knockout mice In 3-month-old WT, Cx40^−/−^, Panx1^−/−^, and Cx40^−/−^Panx1^−/−^ mice Cx43 (green) and N-cadherin (N-Cad, orange) localization was assessed by immunofluorescence in the left (**A**) atria and (**B**) ventricles. Western blots of Cx43 and N-cadherin in atria (**C**) and ventricle (**D**) lysates revealed no difference in the abundance of these junctional proteins between mouse genotypes (*n*=3). Nuclei (**A**,**B**) were stained with Hoechst dye. Arrows and arrowheads in (**A**,**B**) denote Cx43 gap junction plaques and N-cadherin at the ICDs. Dashed lines in the Western blots shown in (D) reflect using the mirror image of original Western blot to maintain consistent loading order of samples. Scale bar = 20 µm.

Despite the lack of fibrosis, cardiac hypertrophy may also be accompanied by connexin expression remodeling [51], thus WT, Cx40^−/−^, Panx1^−/−^, and Cx40^−/−^Panx1^−/−^ hearts were examined for molecular anomalies at the intercalated discs (ICDs) which may manifest as changes in Cx43 location. However, the distribution of Cx43 gap junctions and N-cadherin at the ICDs in the atria (Figure 6A) and ventricles (Figure 6B) of Cx40^−/−^, Panx1^−/−^, and Cx40^−/−^Panx1^−/−^ mice were comparable with WT. Immunoblotting further revealed that the levels of Cx43 and adhesion molecule N-cadherin were similar in atria and ventricle tissues of knockout and WT mice (Figure 6C,D). These results indicate that the loss of Cx40, Panx1, or both has no detrimental effects on common junctional components found at the ICD.

### Cx40^−/−^Panx1^−/−^ mice phenocopy the cardiovascular status of Cx40^−/−^ mice

**Figure 7:**
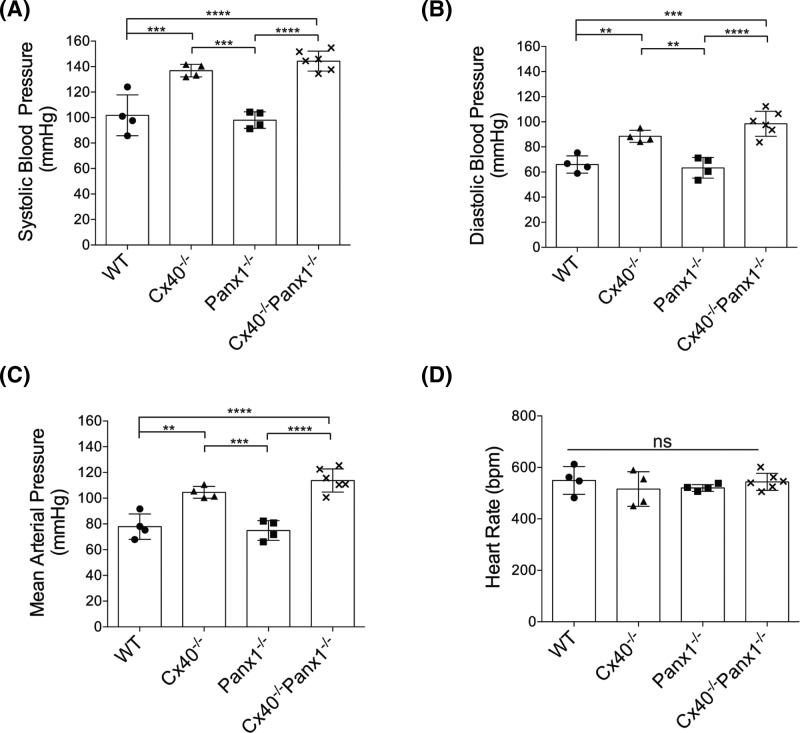
Cx40^−/−^ and Cx40^−/−^Panx1^−/−^ mice are hypertensive Millar pressure catheter recordings of arterial blood pressure revealed that Cx40^−/−^ and Cx40^−/−^Panx1^−/−^ mice have significantly higher (**A**) systolic, (**B**) diastolic, and (**C**) mean arterial blood pressure than WT and Panx1^−/−^ mice (*n*=4–6). (**D**) Mean heart rate is similar amongst all four genotypes (*n*=4–6). **, *P*<0.01; ***, *P*<0.001, ****, *P*<0.0001.

**Figure 8:**
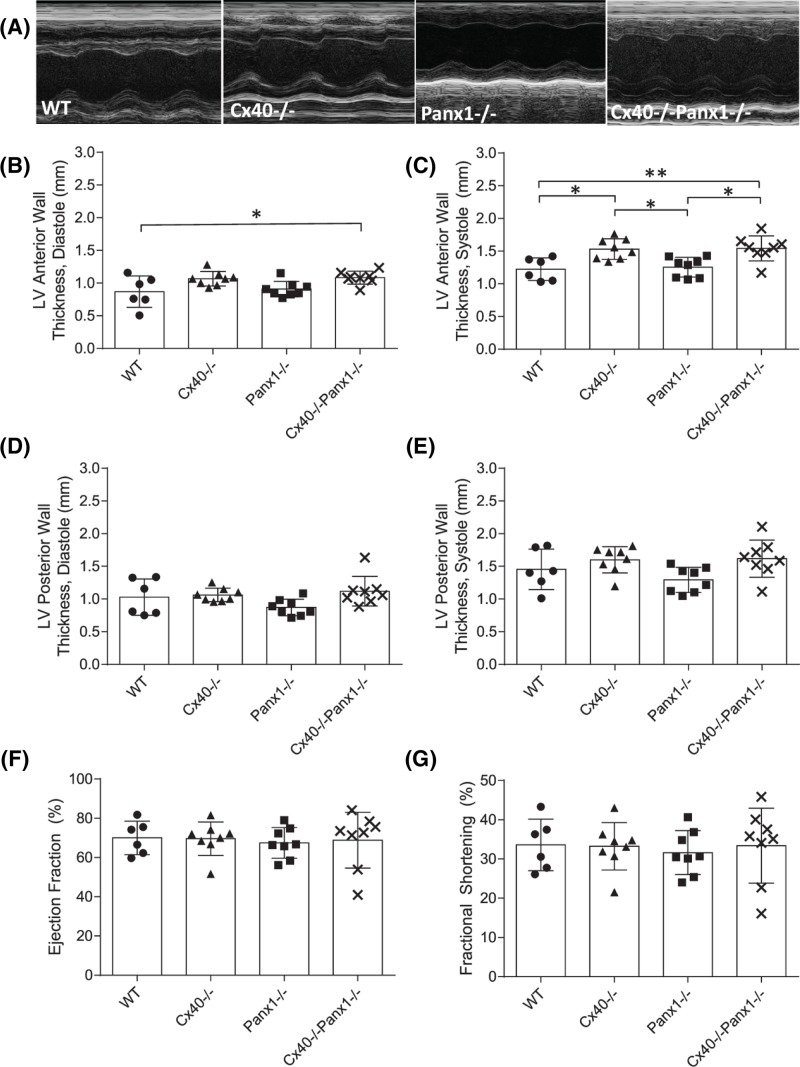
Left ventricular contractility is conserved in Cx40^−/−^ and Cx40^−/−^Panx1^−/−^ mice, despite cardiac hypertrophy and hypertension Representative echocardiographic M-Mode images (**A**). Left ventricular anterior and posterior wall dimensions during systole and diastole (**B**–**E;** *, *P*<0.05, **, *P*<0.01). Left ventricular diameter during systole and diastole were similar across mouse genotypes as measured using M-Mode echocardiography, thus calculated left ventricular ejection fraction (**F**) and fractional shortening (**G**) were comparable amongst WT, Cx40^−/−^, Panx1^−/−^, and Cx40^−/−^Panx1^−/−^ mice (*n*=6–8).

Cardiac hypertrophy is frequently associated with hypertension, and has been previously reported in the Cx40^−/−^ mouse, thus arterial blood pressure was assessed in WT, Cx40^−/−^, Panx1^−/−^, and Cx40^−/−^Panx1^−/−^ mice via carotid pressure transducer catheterization. Systolic, diastolic, and mean arterial pressure were significantly elevated in Cx40^−/−^ and Cx40^−/−^Panx1^−/−^ mice compared with WT and Panx1^−/−^ mice (Figure 7A–C, *P*<0.0001–0.01). Interestingly, heart rates were found to be similar across all genotypes (Figure 7D). We further investigated *in vivo* cardiac functional status in WT, Cx40^−/−^, Panx1^−/−^ and, Cx40^−/−^Panx1^−/−^ mice using M-mode echocardiography (Figure 8). Ejection fraction and fractional shortening, along with systolic and diastolic left ventricular internal diameter (LVID) was similar across all genotypes, indicating conserved cardiac function in knockout mice (Figure 8F, G).

**Figure 9:**
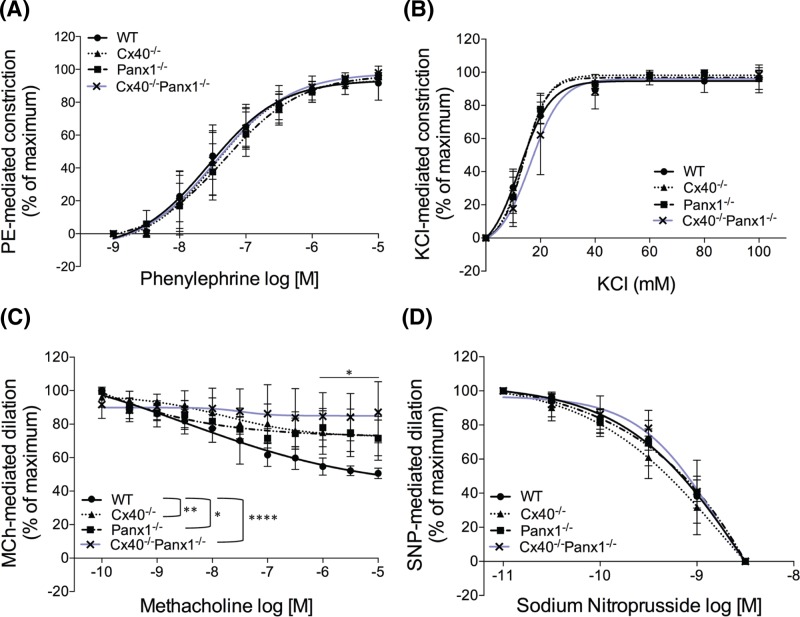
Endothelium-dependent vasodilatory responses are blunted in aortic segments of knockout mice, where responses to vasoconstriction are unaltered Dose–response curves to (**A**) PE, (**B**) KCl, (**C**) MCh, and (**D**) SNP were performed to assess thoracic aorta contractility and dilation, amongst 2–4-month-old WT, Cx40^−/−^, Panx1^−/−^, and Cx40^−/−^Panx1^−/−^ mice. MCh-evoked vasodilatory responses of aortic segments from knockout mice were compromised compared with WT mice at concentrations 1 µM and greater. * represents difference from WT, *P<0.05; **P<0.01; ****P<0.0001. Error bars = SEM.

To determine whether SMC and EC vasomotor signaling mechanisms were affected by the ablation of Cx40 and/or Panx1, isometric tension experiments were performed on aortic rings using a wire myograph. PE and KCl were used to assess SMC-mediated vasocontractile responses, and MCh and SNP were used to measure EC-mediated and EC-independent vasodilatory responses, respectively. Contractile responses to PE and KCl were similar across WT and knockout mice (Figure 9A,B). Conversely, MCh-mediated vasodilation was significantly decreased in Cx40^−/−^, Panx1^−/−^, and Cx40^−/−^Panx1^−/−^ mice compared with WT after the addition of 1 µM or higher of agonist (Figure 9C, *P*<0.0001–0.05). Nevertheless, EC-independent vasodilation in response to SNP was unaltered amongst all four genotypes (Figure 9D).

### Ablation of Panx1 in Cx40^−/−^ mice exacerbates renin dysregulation and renin-producing cells’ displacement

**Figure 10:**
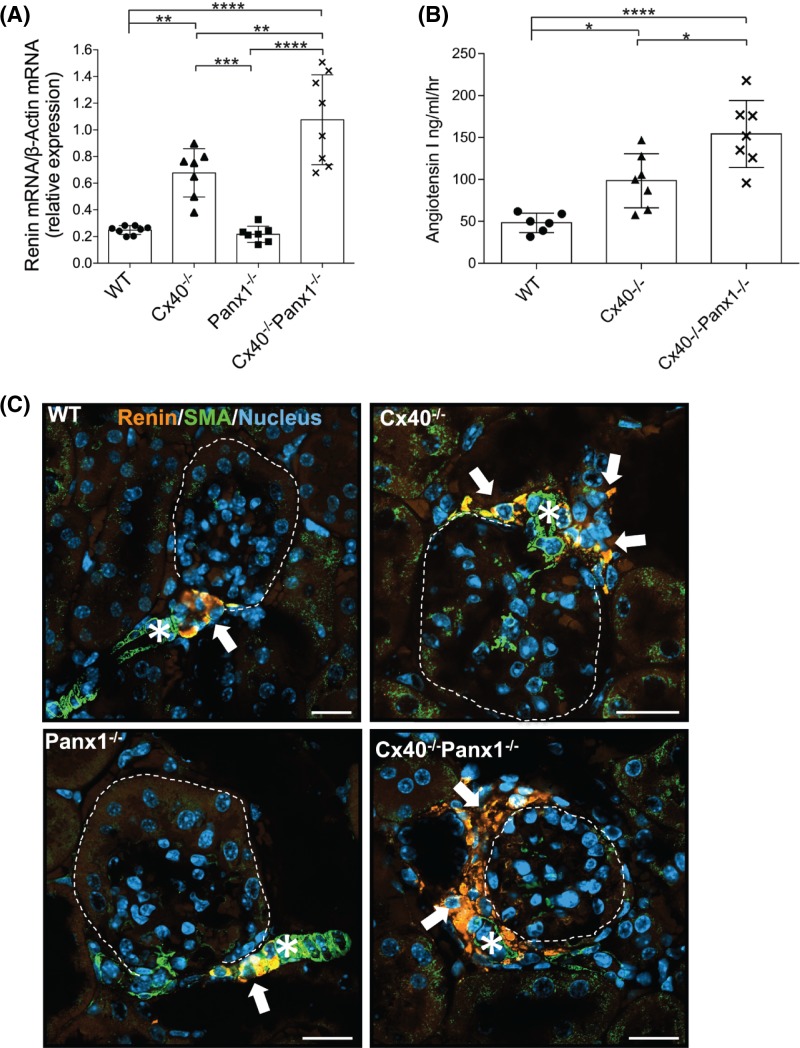
Ectopic renin-producing cell localization, and elevated kidney renin mRNA expression and plasma renin activity in Cx40^−/−^ and Cx40^−/−^Panx1^−/−^ mice (**A**) Kidney renin mRNA levels were elevated in Cx40^-/-^ and Cx40^−/−^Panx1^−/−^ mice compared with WT and Panx1^−/−^ mice (*n*=7–8). (**B**) Plasma renin activity was elevated in Cx40^−/−^ mice compared with WT, where plasma renin activity levels of Cx40^−/−^Panx1^−/−^ mice surpassed that of Cx40^−/−^ mice (*n*=6–7). (**C**) Populations of renin-producing cells (arrows) were observed in the periglomerular space in Cx40^−/−^ mice and to a greater extent in Cx40^−/−^Panx1^−/−^ mice, compared with normal juxlaglomerular positioning at the afferent arteriole proximal to the glomerulus (asterisks) in WT and Panx1^−/−^ mice. *P<0.05; **P<0.01; ***P<0.001; ****P<0.0001. Scale bar = 20 μm.

Previous studies characterizing the Cx40^−/−^ mouse have reported a perturbed negative feedback of renin secretion, despite exhibiting severe hypertension [13,52]. Thus to determine whether the ablation of Panx1 exacerbates dysregulation of renin secretion in the absence of Cx40, we assayed kidney mRNA and plasma renin activity in Cx40^−/−^Panx1^−/−^ mice, and compared expression levels with WT, Cx40^−/−^ and Panx1^−/−^ mice. As expected, tissue-level expression of kidney renin mRNA was elevated in Cx40^−/−^ mice (172 and 213% greater than WT and Panx1^−/−^, respectively, *P*<0.001–0.01); however, Cx40^−/−^Panx1^−/−^ mice demonstrated elevated renin expression compared with all other genotypes (59% increase relative to Cx40^−/−^, *P*<0.01). Interestingly, Panx1^−/−^ mice exhibited kidney renin mRNA expression levels similar to WT mice (Figure 10A). To investigate plasma renin levels, we assessed the generation of angiotensin-1 substrate via ELISA using a renin activity assay (index of plasma renin activity). Cx40^−/−^ mice demonstrated a 104% increase in plasma renin activity compared with WT (*P*<0.05), as expected; however, plasma renin activity levels of Cx40^−/−^Panx1^−/−^ mice surpassed that of Cx40^−/−^ by 57% (Figure 10B, *P*<0.05). Further investigation of renin expression at the level of the glomeruli was assessed using confocal fluorescence microscopy (Figure 10C). In WT and Panx1^−/−^ mice, renin expression was confined to cells located in the wall of the afferent arteriole of the glomerulus. Interestingly, Cx40^−/−^, and to a greater extent Cx40^−/−^Panx1^−/−^ mice, exhibited larger populations of renin-expressing cells beyond the juxtaglomerular region and into the extraglomerular interstitial space (Figure 10C).

## Discussion

Both tissue-specific and global Panx1 knockout mice have been engineered in recent years to assess the roles of these large-pore channels in normal physiology and disease [53,54]. Since the generation of these genetic models, Panx1 has emerged as an important player in cardiovascular physiology. This was highlighted when Petric et al. [32] reported high levels of Panx1 within murine atria, and that Panx1 ablation led to increased susceptibility to atrial fibrillation. As well, Panx1 has been implicated in α1-adrenoreceptor mediated vasoconstriction [7], blood pressure regulation [28], and renal function [55]. Interestingly, the gap junction protein Cx40 shares many of these characteristics including its abundance in the atria, and involvement in cardiac, vascular, and renal function [56,57]. Thus, we generated the first Cx40^−/−^Panx1^−/−^ mouse to assess the redundant, compensatory, or exclusive roles that Cx40 and Panx1 may have in mediating cardiovascular physiology. Cx40^−/−^ and Cx40^−/−^Panx1^−/−^ mice exhibited elevated cardiac hypertrophy and were severely hypertensive compared with Panx1^−/−^ and WT mice, despite no observable decrements in *in vivo* cardiac function as assessed by echocardiography. Furthermore, Cx40^−/−^, Panx1^−/−^, and Cx40^−/−^Panx1^−/−^ mice all demonstrated impairments in EC-mediated vasodilation of aortic segments to MCh as compared with WT. Interestingly, kidney renin mRNA expression and plasma renin activity were greatest in Cx40^−/−^Panx1^−/−^ mice compared with Cx40^−/−^, which already exceeded that of WT. These results may be due to the presence of ectopically located renin-producing cell populations within the extraglomerular interstitial space. Based on our findings, Cx40 and Panx1 may play integrated roles in supporting renin autoregulatory loops, but with regard to many features of the cardiovascular system, mice lacking both Cx40 and Panx1 phenocopy mice lacking only Cx40.

### Loss of both Cx40 and Panx1 does not impact fetal and adult development

Generation of the novel Cx40^−/−^Panx1^−/−^ mouse is the first systemic knockout mouse model in which a connexin and pannexin channel type have been concomitantly ablated. However, in one other study Cx43 and Panx3 were conditionally ablated in the bone to assess the potential complementary role these channel proteins have in skeletal development [58]. Due to the prevalence of Cx40 and Panx1 throughout the cardiovascular system, we anticipated that Cx40^−/−^Panx1^−/−^ mice would have a reduced ability to survive. Intriguingly, Cx40^−/−^Panx1^−/−^ mice were fertile and viable, and generated similar litter sizes to the other genotypes. A previous report describes a mild pre/postnatal death in the Cx40^−/−^ mouse that was attributed to inadequate propagation within the cardiac conduction system [59], however this was not the case in our study as Cx40^−/−^ mice only had statistically insignificant 4% fewer offspring compared with WT. Furthermore, over the 1-year period of tracking mouse weight and morphology, there was no indication of developmental or lifespan abnormalities amongst genotypes. Since the Cx40^−/−^ and Cx40^−/−^Panx1^−/−^ mice exhibit cardiac hypertrophy, hypertension, and renin dysregulation, reductions in lifespan may become more apparent in an aged cohort of mice; however, this remains to be investigated.

### Cardiac hypertrophy and cardiomyocyte enlargement in Cx40^−/−^ mice is not exacerbated by the ablation of Panx1

One of the most striking features of mice lacking Cx40 or both Cx40/Panx1 was their hypertrophic hearts. Arterial hypertension is the most common cause of pressure overload within the heart which often leads to pronounced cardiac hypertrophy, with the LV being the most severely affected. This phenomenon has been previously reported in Cx40^−/−^ mice where the authors describe an increase in heart weight [40]. Here, we observed cardiac hypertrophy in young and adult Cx40^−/−^ mice and confirmed that it was primarily due to the enlargement of cardiomyocytes. Contrary to our expectations, the phenotype observed in mice lacking both Cx40 and Panx1 was not exacerbated. This suggests that cardiac hypertrophy in the Cx40^−/−^Panx1^−/−^ mouse is primarily due to the absence of Cx40 channels.

### Cardiac hypertrophy in Cx40^−/−^ and Cx40^−/−^Panx1^−/−^ mice is not compounded by cardiac fibrosis or dysregulation of junctional proteins

Hypertrophy-induced myocardial remodeling is characterized by augmented interstitial fibrosis, connexin lateralization, and cytoskeletal remodeling which are alterations that often lead to an arrhythmogenic phenotype and heart failure [60]. Across all genotypes, minimal interstitial fibrosis was identified and there were no significant changes in collagen 1 or fibronectin expression in either the left atria or ventricle, suggesting that cardiac hypertrophy did not result in acute fibrosis. Likewise, the distribution of Cx43 and N-cadherin at the ICD was unaltered in knockout mice, as was the myofibril arrangement. Taken together, these findings indicate that cardiac hypertrophy of Cx40^−/−^ and Cx40^−/−^Panx1^−/−^ mice may lead to myocardial remodeling; however, hearts from Cx40^−/−^ and Cx40^−/−^Panx1^−/−^ mice do not yet possess augmented extracellular matrix protein depositions or alterations in ICD integrity that would lead to heart failure. Because hypertrophy is often an adaptive physiological process in response to increases in cardiac workload, and is dynamically regulated, we speculate that in later life, elevated fibrosis, gap junction, and cytoskeletal remodeling may occur in the Cx40^−/−^ and Cx40^−/−^Panx1^−/−^ mice [61], but this is yet to be demonstrated.

### Cx40^−/−^ and Cx40^−/−^Panx1^−/−^ mice are similarly hypertensive, demonstrate preserved ventricular function, and exhibit perturbed EC-mediated vasodilation

Cardiac hypertrophy is often a comorbidity of hypertension, thus arterial blood pressure was assessed in all genotypes. Previous studies have identified the Cx40^−/−^ mouse to be severely hypertensive compared with WT controls, a condition that results from impairments in nitric oxide dynamics in the vascular wall [9], impaired conducted dilation along arterioles [8] and dysregulation of the renin–angiotensin–aldosterone system [62]. Previous findings of hypotension were reported in the SMC-specific Panx1^−/−^ mouse model [28], thus leading to the speculation that combined ablation of Cx40/Panx1 might result in a reduced blood pressure phenotype. Surprisingly, similar to Cx40^−/−^ mice, it was found that Cx40^−/−^Panx1^−/−^ mice maintain a hypertensive blood pressure status which was not observed in Panx1^−/−^ and WT groups. We further assessed *in vivo* cardiac function using echocardiography and determined that LV contractility was preserved in both Cx40^−/−^ and Cx40^−/−^Panx1^−/−^ mice, despite observed cardiac hypertrophy and hypertension. Since LV internal dimension was similar between genotypes, hypertrophy did not adversely affect LV chamber size, but rather manifested as thickening of the LV wall in both Cx40^−/−^ and Cx40^−/−^Panx1^−/−^ mice to a similar extent. Concerning atrial function, both Cx40^−/−^ and Panx1^−/−^ mice have been documented to be increasingly susceptible to atrial arrhythmias [32,40].

With regard to EC function, EC-mediated vasodilation of aortic segments to increasing concentrations of MCh (muscarinic receptor agonist) was impaired across all knockout models compared with WT. Conversely, we observed no differences in SMC-mediated vasoconstriction of aortic segments to PE (α1 adrenergic receptor agonist) or KCl across all genotypes, nor were there differences in EC-independent vasodilatory responses to SNP. These findings implicate a role for both Cx40 and Panx1 in mediating vasodilatory mechanisms in the EC wall. Previously, both Cx40 and Panx1 have been localized to ECs in conduit arteries, are implicated in modifying vasodilatory mechanisms, and conduit arteries of Cx40^−/−^ and Panx1^−/−^ mice exhibit impaired vasodilatory and conducted vasodilatory responses [8,9,21,63]. Specifically, it has been reported that vascular segments from Cx40^−/−^ mice demonstrate impaired vasodilatory responses to the EC-mediated vasodilator acetylcholine due to reduced nitric oxide production and decreased endothelial nitric oxide synthase protein expression [9]. In addition, conducted vasodilatory responses along intact arteriolar segments in skeletal muscle of Cx40^−/−^ mice are impaired, highlighting the role of Cx40 in the spread of vasodilatory responses throughout the microvasculature and its involvement in EC intercellular communication [8]. Previous studies investigating the role of Panx1 in the vasculature have also demonstrated reduced vasodilatory responses of conduit arteries to the EC-dependent vasodilator acetylcholine in Panx1^−/−^ compared with WT mice [63]. This phenotype was found due to the lack of Panx1-mediated ATP release in these knockout mice attributing to a reduction in the endothelium-derived hyperpolarization-like component of vasodilation [64]. Previous reports have also demonstrated Panx1 to mediate SMC-dependent vasoconstriction responses via α1-adrenergic receptor activation in resistance arterioles using an SMC-specific Panx1^−/−^ mouse [7] or saphenous arteries using a global Panx1^−/−^ mouse [63]. This was not demonstrated in the current study since the systemic ablation of Panx1 did not alter vasoconstrictor responses to PE in aortic vascular segments, which is likely due to SMC phenotypic heterogeneity, and differential SMC and EC ratios amongst these two vascular models. Similar to previous studies investigating vascular phenotypes of Cx40^−/−^ and Panx1^−/−^ mice, the Cx40^−/−^Panx1^−/−^ mouse model demonstrates that indeed Cx40 and Panx1 contribute to EC-mediated regulation of vascular tone, but may do so in a redundant or compensatory manner in co-ordination with other vasoactive mechanisms since the loss of both channels does not further enhance EC dysfunction.

### Loss of both Cx40 and Panx1 further disrupts renin homeostasis and renin-cell localization compared with Cx40^−/−^ mice

Previous studies using the systemic Cx40^−/−^ mouse have identified a crucial role for Cx40 in regulating renin secretion and negative feedback inhibition, as these mice exhibit adverse renin secretion and renin-producing cell distribution compared with WT mice [48,65–67]. In the current study, despite observing similar levels of hypertension and EC-mediated dysfunction between Cx40^−/−^ and Cx40^−/−^Panx1^−/−^ mice, kidney renin expression and plasma renin activity were enhanced in mice lacking both Cx40 and Panx1 compared with Cx40^−/−^ mice. This also correlated with Cx40^−/−^ and Cx40^−/−^Panx1^−/−^ mice exhibiting elevated kidney renin mRNA levels compared with Panx1^−/−^ and WT mice, where renin expression in Cx40^−/−^Panx1^−/−^ mice surpassed that of Cx40^−/−^ by 1.6-fold. Plasma renin activity levels were similarly elevated in Cx40^−/−^ mice compared with WT, but again were even greater in Cx40^−/−^Panx1^−/−^ mice. Further examination of renin expression via confocal fluorescence microscopy revealed that Cx40^−/−^ mice exhibit ectopic localization of renin-producing cells outside the juxtaglomerular region and into the extraglomerular intersitium, previously demonstrated in Cx40-deficient mice [48,65–67]. However, renin cell localization exceeding the juxtaglomerular region was demonstrated to a greater extent in the case of Cx40^−/−^Panx1^−/−^ mice, where renin-producing cell populations extended further around the extraglomerular region. Previous work using Cx40^−/−^ mice and renin producing cell-specific Cx40^−/−^ mice have established that Cx40 is essential for controlling the positioning of renin cells in the juxtaglomerular area [48,66–68], as well as intrinsic negative feedback mechanisms of angiotensin II and increased renal pressure on renin [48,62,65,69]. Interestingly, Cx40^−/−^ mice expressing Cx40 only in EC (via crossing with Tie2-Cx40 mice expressing Cx40 solely in EC) did not demonstrate an improved blood pressure or renin phenotype [70]. Blood pressure and renin production and secretion becomes intermediately normalized only when Cx40^−/−^ mice are generated to re-express Cx40 in renin-producing cells, yet this adjustment does not correct the displacement of renin-producing cells within the juxtaglomerular area [66]. Normally, juxtaglomerular renin-producing cells line the afferent arteriole at the entrance of the glomeruli. Here, these cells are positioned to respond to changes in blood pressure and volume via release of renin as glomerular blood flow perfuses the afferent arteriole. When the body experiences extreme homeostatic imbalances that induce chronic renin stimulation (e.g. hypotension, extracellular fluid depletion, low-salt diet), ‘recruitment’ of renin cells can occur. Non-producing renin cells previously responsible for renin secretion during early development along the afferent arteriole re-convert into a renin-producing phenotype in an effort to produce sufficient renin to support blood pressure regulation [71]. In Cx40^−/−^ and Cx40^−/−^Panx1^−/−^ mice however, renin-producing cells appear in abnormal ectopic locations surrounding the glomerulus other than the juxtaglomerular region, and thus renin-responsiveness of the juxtaglomerular apparatus becomes disrupted. This may suggest that Cx40 in conjunction with Panx1 may be necessary for the appropriate homing of renin-producing cells and regulated renin secretion, but within the field, Cx40 deficiency and renin cell displacement is still not understood. In the current study, the global absence of Panx1 alone did not alter renin mRNA expression, suggesting that renin synthesis is unaffected possibly due to the continued presence of Cx40, further suggesting an integrated role of Cx40 and Panx1 at the level of renin-producing cell localization and regulation. Functionally, little is known about the role of Panx1 in the renal system; however, based on its localization within the renal vasculature, and its role in purinergic signaling, it has been previously hypothesized that Panx1 may play a role in pressure natriuresis [55]. Further studies are needed to elucidate the role of Panx1 and its relationship with Cx40 in renin-producing cells, as well as their role in mediating renin synthesis, humoral blood pressure regulation and homing of renin-producing cells to the juxtaglomerular area of the afferent arteriole.

In summary, the generation of the first systemic Cx40/Panx1 knockout mouse revealed cardiovascular phenotypes similar to mice lacking Cx40. Both Cx40^−/−^ and Cx40^−/−^Panx1^−/−^ mice exhibited enlarged hearts and comparable levels of cardiac hypertrophy due to cardiomyocyte enlargement and thickening of the LV wall; an outcome that was not compounded by impaired LV contractility, cardiac fibrosis, or disruption of molecular components at the ICDs. Likewise, both the Cx40^−/−^ and Cx40^−/−^Panx1^−/−^ mice demonstrated similar levels of hypertension, while Panx1^−/−^ mice were normotensive. Assessment of vascular reactivity demonstrated that all knockout genotypes exhibited impaired EC-mediated vasodilation compared with WT, highlighting the functional role of Cx40 and Panx1 in the EC layer of the vasculature. Most interestingly, Cx40/Panx1 ablated mice had kidney renin expression levels and plasma renin activity levels that exceed Cx40 null mice, which coincided with aberrant ectopic localization of renin-producing cell populations to the extraglomerular interstitium. These novel findings uncover a potential integrative relationship between Cx40 and Panx1 in the juxtaglomerular apparatus of the kidney and regulation of renin.

## Abbreviations

Cx: connexin
EC: endothelial cell
ICD: intercalated disc
LV: left ventricle
LVID: left ventricular internal diameter
LVIDd: LV internal end-diastolic dimension
MCh: methacholine
Panx: pannexin
PBST: PBS plus Tween-20
PE: phenylephrine
SA: sinoatrial
SMC: smooth muscle cell
SNP: sodium nitroprusside
WT: wild-type
